# Time to Organize the Bioinformatics Resourceome

**DOI:** 10.1371/journal.pcbi.0010076

**Published:** 2005-12-30

**Authors:** Nicola Cannata, Emanuela Merelli, Russ B Altman

We will be witnessing the birth of the artificial, or in-silico, scientist. —J. D. Wren [1]

The field of bioinformatics has blossomed in the last ten years, and as a result, there is a large and increasing number of researchers generating computational tools for solving problems relevant to biology. Because the number of artifacts has increased greatly, it is impossible for many bioinformatics researchers to track tools, databases, and methods in the field—or even perhaps within their own specialty area. More critically, however, biologist users and scientists approaching the field do not have a comprehensive index of bioinformatics algorithms, databases, and literature annotated with information about their context and appropriate use. We suggest that the full set of bioinformatics resources—the “resourceome”—should be explicitly characterized and organized. A hierarchical and machine-understandable organization of the field, along with rich cross-links (an ontology!) would be a useful start. It is likely that a distributed development approach would be required so that those with focused expertise can classify resources in their area, while providing the metadata that would allow easier access to useful existing resources.

The growth of bioinformatics can be quantified in many ways. The Intelligent Systems for Molecular Biology Meeting began in 1993, and numerous other meetings have been established. The International Society for Computational Biology (ISCB) was formed in 1995, and recent membership numbers have reached 2,000. The field has gone from having one or two journals to having more than a dozen—if one considers “-omics” (i.e., subjects relating to high-throughput functional genomics, where computation plays a central role) and the emerging field of systems biology. Because bioinformatics has a strong element of engineering, the creation and maintenance of tools provide value only insofar as they are used. These tools may be databases that hold biological data, or they may be algorithms that act on this data to draw inferences. Access to these artifacts is currently uneven. Of course, the published literature is the archival resting place for the initial description of these innovations, but it only contains a snapshot of most tools early in their lifetime. The literature does not use any standard classification system to describe tools, so the sensitivity of searches for specific functions is not generally high. Indeed, the bibliome itself is idiosyncratically organized, and finding the right article is often like searching for a needle in a haystack [2]. Finally, the published literature does not contain reliable references to the location and to the availability of most bioinformatics resources [3,4]. One could also argue that Google (http://www.google.com) provides adequate access to tools based on keyword searching [5]. However, the lack of standard terms makes sensitive and specific searches difficult. In addition, most search hits confound papers, Web sites, tools, departments, and people in a manner that makes extracting useful information very difficult.

Recognizing this limitation, there have been some grassroots attempts to organize the bioinformatics resourceome. Among the most famous are the “archaeological” Pedro's List—a list of computer tools for molecular biologists (http://www.public.iastate.edu/~pedro/research_tools.html)—and the Expasy Life Sciences Directory, formerly known as the Amos's WWW links page (http://www.expasy.org/links.html). The Bioinformatics Links Directory (http://www.bioinformatics.ubc.ca/resources/links_directory/) today contains more than 700 curated links to bioinformatics resources, organized into eleven main categories, including all the databases and Web servers yearly listed in the dedicated *Nucleic Acids Research* special issues [6]. The National Center for Biotechnology Institute has tried to make access to its suite of tools transparent, with moderate success. Many Web sites can be found listing “useful sites,” especially concerning special interest or limited topics (e.g., microarrays, text mining, and gene regulation). But all of these efforts are limited by the difficulty in maintaining currency and by the lack of a uniformly recognized classification scheme. Yet our colleagues in bioinformatics and biology are constantly asking about the availability of tools or databases with certain characteristics. The lack of a useful index, thus, routinely costs time and opportunities. In addition, there is no “peer-review” system for bioinformatics tools so that the most useful ones can be highlighted by happy users. A secure and reliable system for rating (similar to that used by Amazon.com, for example) would also be an important prerequisite.

An “ontology” is a specification of a conceptual space, often used by computer programs. The field of ontology engineering has matured in the last 20 years, making fundamental contributions in computer science and establishing applications in biology. The success of the Gene Ontology Project (it is used by multiple model organism databases, and is used to annotate high-throughput data routinely [8]) is one example of an ontology that was developed for the narrow purpose of supporting comparative genomics, but which has found a multitude of other uses. A primitive bioinformatics-specific ontology is available in Google Directory (http://directory.google.com/Top/Science/Biology/Bioinformatics), assembled in the collaborative Open Directory effort (http://www.dmoz.org), but it, too, mixes all different classes of objects (personal Web sites, organization Web sites, databases, and tools) in a way that is not transparent. It seems clear that a well-organized and intuitive ontology of bioinformatics resources would provide a very valuable framework on which a fully distributed system of registration and annotation of biology-related computational resources could be constructed. The Transparent Access to Multiple Bioinformatics Information Sources (TAMBIS) [9] work was a bold attempt to describe bioinformatics concepts, including resources, using formal description languages. Unfortunately, it has not been widely used, perhaps because it was ahead of its time or because the underlying knowledge representation techniques are somewhat sophisticated and complex.

*In the foreseeable future the web of links between documents, databases, and programs can provide a new level of interaction among scientific communities.* —J. Hendler [10]

Ontologies are important, but their use is often hindered by the lack of “killer apps” for using them. It is often unclear how to exchange information about ontologies, and how to link them to other resources on the Web. Emerging technologies that contribute important infrastructures to the resourceome are represented by the semantic Web and Web services. It is now possible to have standardized descriptors of Web resources, using an ontology, in order to “publish” the availability of tools or simply to announce their existence. Thus, the vision for using an ontology to support the resourceome becomes clear: each individual who has created or who is maintaining a resource uses a standard ontology to describe the basic features of that particular resource using the semantic Web, and these are automatically included in a distributed index of resources. Thus, the index is created by querying the semantic net for descriptions of all available tools, which can then be registered and updated on a regular basis. The development of a browser for this index could be the final step (or “killer app”) in building a self-sustaining, distributed index of bioinformatics resources. Adoption of agent technology may be helpful in overcoming the inherent complexity of this challenge [11].

We believe that the need for a bioinformatics resourceome project and the technical requirements for it are both present. We therefore urge the community to come together to start the process of creating a simple distributed system for describing resources, announcing their availability, and presenting this information to biologists and bioinformaticians in an easy-to-navigate manner. The World Wide Web Consortium already launched its first workshop on Semantic Web for Life Sciences, bringing together more than 100 participants from academia, industry, and international organizations. Another important event is the recent creation of the National Center for Biomedical Ontology (http://www.bioontology.org).

The initial steps toward a bioinformatics resourceome are clear. First, an overall ontology with the high-level concepts (algorithms, databases, organizations, papers, people, etc.) must be created, with a set of standard attributes and a standard set of relations between these concepts (e.g., people *publish* papers, papers *describe* algorithms or databases, organizations *house* people, etc.). The initial ontology should be compact and built for distributed collaborative extension. Second, a mechanism for people to extend this ontology with subconcepts in order to describe their own resources should be designed. The precise location of a tool within a taxonomy is not critical—the author will place it somewhere based on the location of similar/competing resources or based on a best-informed guess. Others may create links to the resource from other appropriate locations in the taxonomy in order to ensure that competing interpretations of the appropriate conceptual location for the resource are accommodated. Third, the formats for the ontologies and the resource descriptions should be published so enterprising software engineers can create interfaces for surfing, searching, and viewing the resources. The resulting distributed system of resource descriptions would be extensible, robust, and useful to the entire biomedical research community.

Who can take leadership in this effort? We believe that a coalition of publishers with an open-access ethic, funding agencies, and scientists who want to contribute to an improved computational infrastructure for biomedicine would be most effective. Companies with an interest in cost-effective research and development may also want to be involved. Most likely, a small group of devoted scientists with both biological domain knowledge and understanding of semantic Web technologies must take the lead. A critical mass of resources must be indexed so that the value of the effort can be assessed. Most likely, the initial indexing will not include all possible resources, but rather algorithms and databases. The community can decide later if Web sites, publications, people, and institutions should also be indexed. The system should also include from the start a capability for routinely evaluating sites for availability (no 404s!). There is increasing discussion of the requirements and technologies for the resourceome at bioinformatics conferences, including Intelligent Systems for Molecular Biology (http://ismb2006.cbi.cnptia.embrapa.br), Pacific Symposium on Biocomputing (http://psb.stanford.edu), and others (see http://www.iscb.org).

## References

[pcbi-0010076-b001] Wren JD (2004). The emerging in-silico scientist: How text-based bioinformatics is bridging biology and artificial intelligence. IEEE Eng Med Biol Mag.

[pcbi-0010076-b002] Grivell L (2002). Mining the bibliome: Searching for a needle in a haystack?. EMBO Rep.

[pcbi-0010076-b003] Wren JD (2004). 404 not found: The stability and persistence of URLs published in MEDLINE. Bioinformatics.

[pcbi-0010076-b004] Schilling LM, Wren JD, Dellavalle RP (2004). Letter to the editor: Bioinformatics leads charge by publishing more Internet addresses in abstracts than any other journal. Bioinformatics.

[pcbi-0010076-b005] Brin S, Page L (1998). The anatomy of a large-scale hypertextual web search engine. Comput Netw.

[pcbi-0010076-b006] Fox JA, Butland SL, McMillan S, Campbell G, Ouellette BF (2005). The bioinformatics links directory: A compilation of molecular biology web servers. Nucleic Acids Res.

[pcbi-0010076-b007] Berners-Lee T, Hendler J (2001). Publishing on the semantic web. Nature.

[pcbi-0010076-b008] The Gene Ontology Consortium (2000). Gene ontology: Tool for the unification of biology. Nat Genet.

[pcbi-0010076-b009] Stevens R, Baker P, Bechhofer S, Ng G, Jacoby A (2000). TAMBIS: Transparent access to multiple bioinformatics information sources. Bioinformatics.

[pcbi-0010076-b010] Hendler J (2003). Science and the semantic web. Science.

[pcbi-0010076-b011] Berners-Lee T, Hendler J, Lassila O (2001). The Semantic Web. Sci Am.

[pcbi-0010076-b012] Neumann E (2005). A life science Semantic Web: Are we there yet?. Sci STKE.

