# Correction: Time to Organize the Bioinformatics Resourceome

**DOI:** 10.1371/journal.pcbi.0020020

**Published:** 2006-02-24

**Authors:** Nicola Cannata, Emanuela Merelli, Russ B Altman

In *PLoS Computational Biology,* volume 1, issue 7, DOI:  10.1371/journal.pcbi.0010076

Two quotes and reference citations were omitted in error:

“More information needs to be in a form that the machine can ‘understand' rather than simply display” —T. Berners-Lee and J. Hendler [7]

“We invite the life science communities to participate together and begin realizing the semantic Web vision.” —E. Neumann [12]

